# Resting-Exercise Salivary Cortisol Responses: Detecting the Magnitude of Hormonal Change Over Time

**Published:** 2016-01-08

**Authors:** Anthony C Hackney, Travis Anderson

**Affiliations:** Endocrine Section-Applied Physiology Laboratory, Department of Exercise & Sport Science. University of North Carolina, Chapel Hill, North Carolina, USA

**Keywords:** Hormones, Stress, Endocrine, Physiology

## Abstract

This study investigated the validity of salivary cortisol responses to reflect blood cortisol responses relative to the magnitude of change observed over time in the hormone. Male subjects (n=25) conducted four experimental sessions (ES) where blood (B) and saliva (S) were obtained before (PS) and after (PoS) a 30 min resting control, 40%, 60%, and 80% of maximal aerobic capacity (VO2max) exercise ES. B and S specimens were analyzed by standard biochemical procedures. Hormonal concentrations changes were assessed by using absolute delta (DA) values (PoS – PS) and percent change (PC) calculations ((PoS-PS)/PS × 100) for each B and S specimen. Subsequent DA and PC values were correlated (Pearson) for each B-S specimen pairing (n=100; n=25 × 4 ES). Results indicate the magnitude of change (PoS vs. PS) in S cortisol is more valid and strongly associated (p<0.001) with corresponding B changes (the “gold standard”) when expressing the data as delta values using absolute hormonal concentrations as compared to percent change expression.

## Introduction

Stress results in activation of the hypothalamic-pituitary-adrenal (HPA) axis inducing elevated glucocorticoid responses; which in humans is primarily the hormone cortisol. Blood cortisol is studied as a biomarker across a wide variety of scientific disciplines and these responses typically serve as the “gold standard” by which to assess the scale of hormonal reactivity and HPA status. However, in many research situations obtaining blood specimens is not practical due to a variety of issues; such as, the subject population (i.e., children), experimental design, or the invasiveness of the procedure. To this end, salivary cortisol measurement serves as a popular substitute means for assessing hormonal reactivity and the HPA status.

One powerful stimulant provoking the HPA axis and cortisol change is physical exercise. Resistance and endurance-based exercises can cause changes in resting cortisol of 100–500%, provided the exercise intensity is appropriate [1]. Both blood and salivary cortisol levels respond to exercise and evidence supports relatively good agreement between the blood and salivary levels when looking at absolute concentrations at the same point in time (i.e., r^2^ ~50–80%) [2–4].

Many studies involving acute exercise sessions examine hormone changes before and after the session (i.e., pre, post comparisons; repeated measures statistical design) in individual subjects. Hence, these studies are interested in the magnitude of the hormonal change responses due to the effect of exercise [5]. To that end, it appears many studies comparing blood and salivary cortisol responses have examined the associations between absolute hormonal values. That is, cortisol concentrations in blood and saliva collected at the same time point have been simply correlated. A preliminary report from our laboratory suggested this may be a questionable approach for presenting relationship between salivary-blood samples and discerning the magnitude of hormonal change due to “range effect” influences (i.e., statistical phenomena resulting in compressed variance [6,7]. Furthermore, some of these prior exercise studies have limitations that compromise their generalizability; such as, a) small sample sizes, b) no control for diurnal-circadian aspects of the HPA axis, and c) inadequate control for subject prior diet or psychological stressor exposure. In light of these above points, this study was conducted with a purpose of investigating the validity of salivary cortisol responses to reflect blood cortisol responses (especially in response to exercise) relative to the magnitude of change observed over time in the hormone.

## Methods

### Participants

Male subjects (ranges; age 18–30 yr; body mass 66.5 – 78.4 kg, height 166.5–184.5 cm, n=25) who were involved in exercise training activities ≥3 d/wk, ≥60 min/d for the ≥6 months before the study were recruited. Written informed consent was obtained from each subject prior to participation in accordance with Institution Review Board procedures and the Helsinki Declaration. Subject exclusion criteria included a diet chronically low in carbohydrates (CHO; <50% daily intake), a history of hormonal disorders, mental illness, smoking, drug use of any kind, or heightened emotional stress.

### Procedures

Subjects reported to the laboratory on five separate occasions during which they maintained and controlled their diet for the 24 hours before each arrival (eucaloric, >50% CHO, assessed by diet records). For all sessions, subjects reported 4 h postprandial, having consumed no caffeine or alcohol for the previous 8 h.

The specifics of the study protocols are reported in detail elsewhere [8]. In brief, at the first laboratory session, maximal aerobic exercise capacity (VO^2^max; 60.1 ± 5.9 mL/kg/min, X ± SD) was determined using an electrically braked cycle ergometer (Lode, The Netherlands) and a Parvo Medics indirect calorimetry system (Parvo Medics, Park City, Utah, USA). Their next four experimental sessions (ES) consisted of a control rest period, and three 30 min cycling exercise bouts at 40%, 60%, or 80% intensity of VO^2^max. All ES were at the same time of day (±30 min), randomly assigned and separated by a minimum of 72 h.

Subjects began each ES by completing a REST-Q emotional stress questionnaire, if normal scores representative of low stress levels, they rested supine for 30 min; if not they were excused from testing that day and rescheduled [9]. After the rest period, a pre-ES (PS) blood (B) and saliva (S) specimen was obtained (order always B→S). If the ES was exercise, they then performed a 10 min warm-up of light cycle ergometry and stretching, and then began exercising at a cycling workload to elicit 40%, or 60%, or 80% VO^2^max. At the end of 30 min of exercise an immediate post-ES (PoS) B-S specimens were collected. If their ES was the control, the above procedures were repeated, except a ~40 min supine rest was completed by the subjects with B-S collected at corresponding times.

The B and S specimens were collected and treated using standardized clinical procedures described extensively elsewhere [5,8]. Cortisol B (blood serum) was analyzed using radioimmunoassay techniques (Siemens Health Care, Los Angeles, USA) while cortisol S was analyzed using enzyme immunoassays techniques (Salimetrics Inc., State College, PA, USA). All assay procedures were done in duplicate determination and assay coefficients of variance (within and between analysis batches) were required to be less than 10%.

### Statistics

The design of this study resulted in 100 subject visits (n=25 × 4 ES) with B and S cortisol concentrations being measured in both the PS and PoS specimens within each ES. The B and S values were converted and expressed in two forms which are frequently used to depict change [10]. This included; a) calculating an absolute delta value [DA] = PoS − PS; and b) calculating a percent change value [PC] = {{PoS−PS} ÷ PS} × 100. The resulting magnitude of change values (DA, PC) for the match pairs of B and S values were examined for strength of association using Pearson (Pc) correlation analysis (S = independent variable, B = dependent variable). The resulting Pc coefficients for DA and PC were tested for significant differences from one another using the Steiger procedure for correlations from dependent measures [11]. Additionally, the linear regression analyses from the Pc were used to calculate residuals and the Durbin-Watson d statistic used to assess for autocorrelation influence (i.e., since repeated measures were obtained on each subject) [12]. Alpha level was set at 0.05 a priori.

## Results

Cortisol B and S concentration responses at PS and PoS within each of the ES are reported in Table 1.

**Figure 1:**
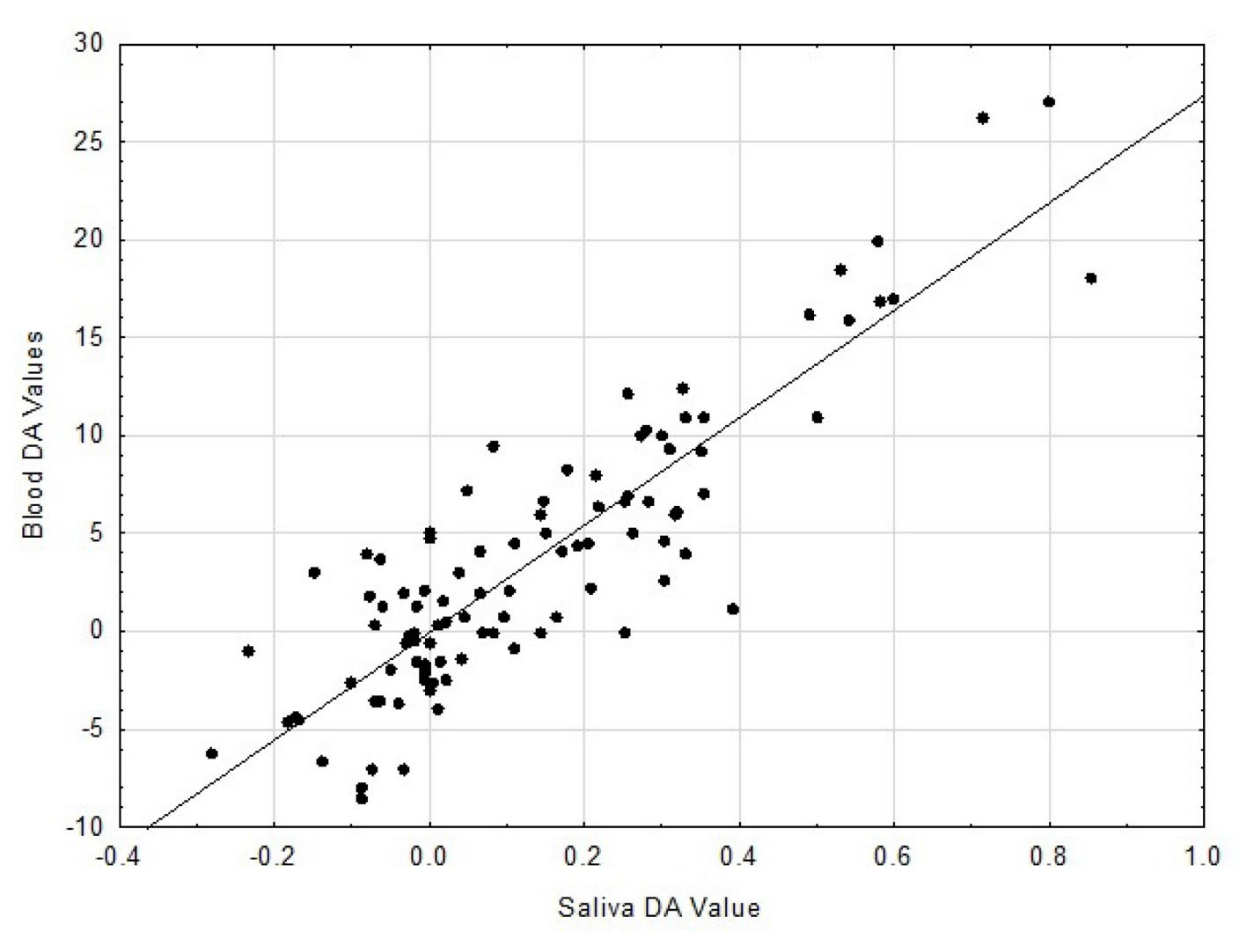
Scatter plot with regression line of DA values for S and B specimens at all ES (r=0.880; n=100; Blood units=μg/dL; Saliva=μg/dL).

**Figure 2:**
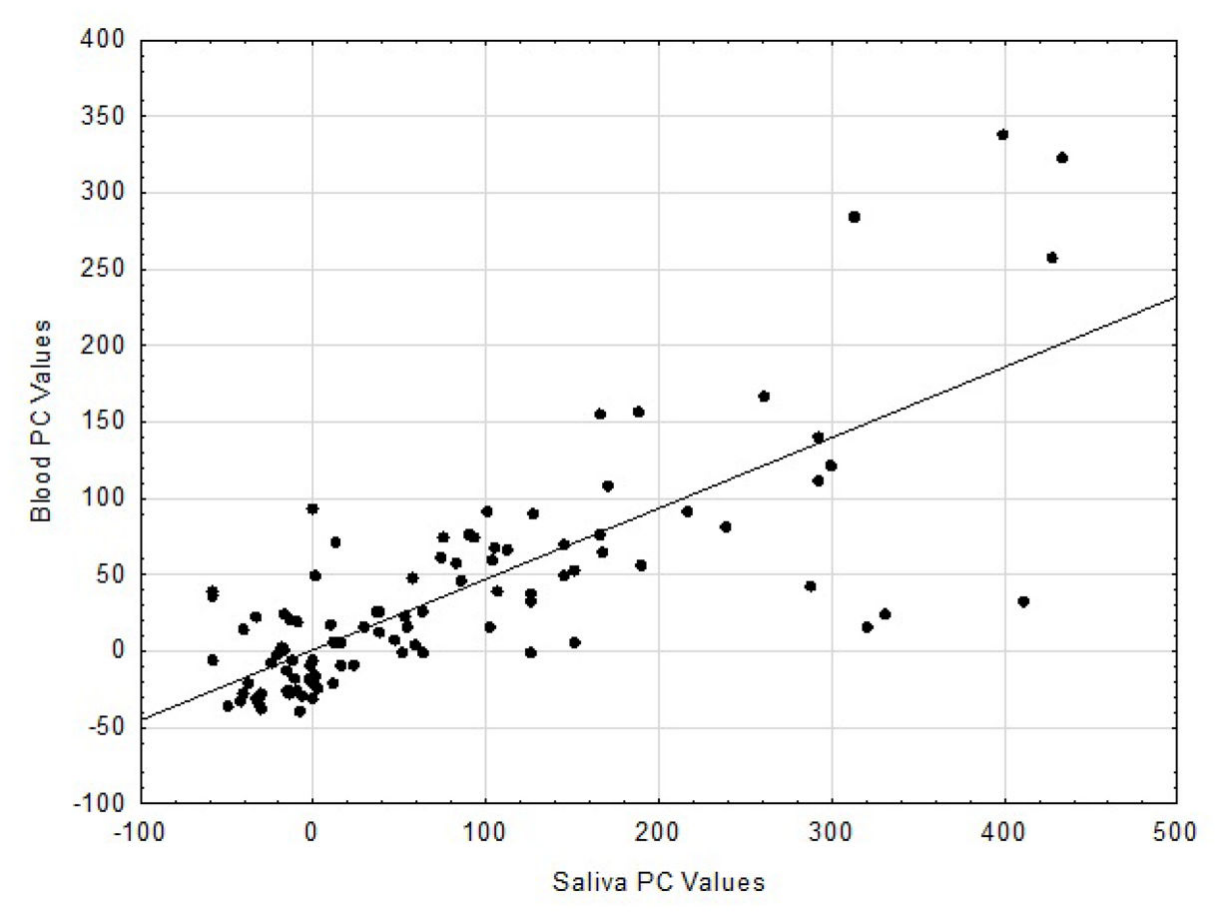
Scatter plot with regression line of PC values for S and B specimens at all ES (r=0.772; n=100; Blood units=μg/dL; Saliva=μg/dL).

All B and S values agree with previous reported findings in the literature [2,3]. Results of the Pc correlational analysis revealed for the DA values an r = 0.880 (p<0.001; see Figure 1) and for the PC values an r = 0.772 (p<0.001, see Figure 2).

**Figure 3:**
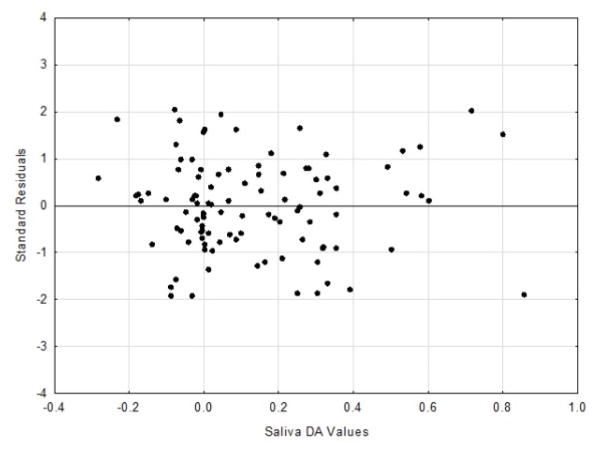
Plot of standardize residuals vs. saliva DA values.

**Figure 4:**
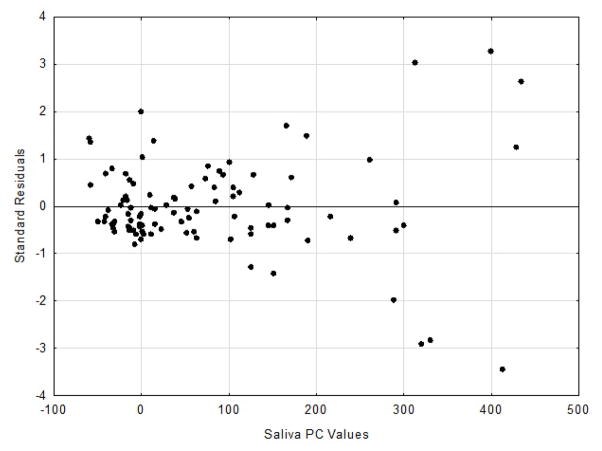
Plot of standardize residuals vs. saliva PC values.

These two coefficients differed significantly from one another (p<0.01). The residuals scatter plots from the respective DA and PC linear regression analyses are shown in Figures 3 and 4.

The DA residual analysis resulted in a Durbin-Watson d value of 2.08, while the PC residual analysis d value was 2.13. The Durbin-Watson d statistic ranges from 0 to 4, with a value of 2.0 or greater indicating no autocorrelation [12].

The VO^2^ responses to the 40%, 60% and 80% exercise are in agreement with previous findings [8,13]. Additionally, the responses between subjects were relatively homogenous and have been reported elsewhere [14].

## Discussion

Our purpose was to examine the validity of S cortisol responses to reflect B cortisol responses relative to the magnitude of change observed over time, with a special emphasis on exercise induced changes. This focus was chosen because many exercise endocrinology studies are interested in the degree of hormonal change before and after a period of time (i.e., an experimental treatment or intervention) and hence use repeated measures statistical designs [5].

All the correlation coefficients obtained were highly significant with the respective associations accounting for 77.4 8% (DA) and 59.6% (PC) of the variance between the S and B specimen pairs. Using the criteria of Hopkins, the effect size for both the coefficients was “very large” (<0.9>0.7, range for large classifications) [10].

The level of association for the current coefficients agree with a number of previous published findings [2,3,8], but not all, as Sumioka and associates reported lower levels of coefficients [15]. However, as noted many research studies have correlated the absolute hormonal concentrations obtained for S and B at the same point in time and have not consistently looked at the strength of the association for changes over time (as was done presently in this study). This difference in protocols between the present study and the other ones just noted, obviously presents limitations in our ability to make direct comparisons between studies.

In assessing correlations “predictive validity”, residuals from the bivariate regression analysis can be calculated and used as a means of accessing accuracy [4,13,16]. In examining the DA residuals, based upon the criteria of Hocking, the scatter plot results can be classified as unbiased and essentially homoscedastic (uniformed residual variance across the range of the independent variable) [17]. Conversely the PC residuals can be classified as unbiased and more heteroscedastic (non-uniformed residual variance across the range of the independent variable) [17]. The assumption of homoscedasticity (i.e., same variance) is central to linear regression models [10]. Homoscedasticity describes a situation in which the error term (i.e., the “noise” or random disturbance in the relationship between the independent variables and the dependent variable) is the same across all values of the independent variables. Heteroscedasticity occurs when the size of the error term differs across values of an independent variable [10].

The intent of linear regression is to minimize residuals and in turn produce the smallest possible standard errors; but when heteroscedasticity is present the cases with larger disturbances have more “pull” than other observations. Thus, the current residuals analysis point to the DA data being more appropriate (i.e., valid) for use in determining the magnitude of change, when assessed with the correlative – linear regression approach of comparison. It could be argued that a Bland-Altman plot might lead to more clarity on the issue of validity than the regression and residuals; but Hopkins, a leading statistician in the Exercise Sciences, has argued that the Bland-Altman plot has a bias that makes it inappropriate for use and leads to erroneous interpretations [17]. Specifically, Hopkins points to an “artefactual bias arises in a Bland-Altman plot of any measures with substantial random error” [17].

Data conversion or transformation, such as calculating the percent change (PC), is usually done to adjust for a non-normal distribution within a data set (a highly likely occur in hormonal endocrine measurements) so as to allow for a subsequent appropriate use of inferential analysis (i.e., ANOVA) [7,10]. This is an accepted practice in exercise endocrinology research [5,16]. Yet, in the context of the correlation analysis the current findings reveal the strength of association between the independent-dependent variables when assessing magnitude of change supports the use of a simple delta conversion expression of the data. In the present data when the Kolmogorov-Smirnov “Goodness-of-Fit” test was applied, it revealed the S and B data sets (concentration values) did not violate the assumption of normal distribution, and hence did not need a conversion-transformation (although, we chose to do it as a means of illustrating a point). It is suggested that researchers use the “Goodness-of-fit” test or other such statistical procedures to determine distribution normality first and not just automatically convert-transform data unless a violation of the assumption of normal distribution are detected [4].

There are several delimitations - limitations to this study. First is the use of only one hormone (cortisol), as there are a multitude of potential endocrine measures that could be assessed in B and S. But, cortisol is one of the most frequently assessed hormones in exercise studies due to its metabolic role as well as stress reactivity responsiveness [16]. Secondly, S cortisol is an expression of free cortisol levels, while B cortisol represents total cortisol (free + bound hormone) [16,17]. Ideally the B specimens would also be measurements of free cortisol, but this is a rare clinical measurement and the vast majority of reported research uses total cortisol as an assessment. Also, the total number of subjects, twenty-five, could be considered relative small within a correlational study. To try and overcome this point we used the pair of responses across all of the ES, hence allowing the sample size to be much larger (n=100). Finally, it could be argued that the Pc analysis is biased due to the inclusion of repeated measurements from the same subjects (autocorrelation). But, the results of the Durkin-Watson analysis suggestion that any autocorrelation bias was extremely minimal in the current data as based upon the residual analysis [12].

In conclusion, if researchers are interested in expressing the magnitude of change in saliva cortisol before and immediately after exercise, the conversion of data to delta values using absolute hormonal concentrations is recommended; N.B., provided there is a normally distributed data set. Expressing the data in this fashion shows the strongest association to the “gold standard”, blood responses. The large sample size and tightly controlled experimental conditions in executing this study strengthen the soundness of this conclusion, but additional research is needed on other hormones in order to test the generalizability of these findings across other endocrine measures.

## Figures and Tables

**Table 1:** Hormonal concentration for cortisol in blood and salivary specimens (n=25; X ± SD).

Specimen	Experimental Session (ES)	Pre-session (PS)	Post-Session (PoS)
Blood (B) (μg/dL)	Control	11.6 ± 4.3	9.6 ± 3.4
	40% VO^2^max	13.7 ± 6.5	14.2 ± 4.6
	60% VO^2^max	13.4 ± 4.1	17.3 ± 4.7*
	80% VO^2^max	12.3 ± 3.7	23.7 ± 6.7*
Saliva (S) (μg/dL)	Control	0.24 ± 0.25	0.24 ± 0.13
	40% VO^2^max	0.27 ± 0.17	0.26 ± 0.15
	60% VO^2^max	0.22 ± 0.10	0.33 ± 0.13*
	80% VO^2^max	0.19 ± 0.08	0.52 ± 0.24*

* Repeated measures ANOVA for difference within respective B and S specimens at PS vs. PoS.
